# Diseases of Women, and Obstetrics

**Published:** 1859-05

**Authors:** 


### DISEASES OF WOMEN, AND OBSTETRICS.

XII.—*Observations on the Use of the Tampon.* By H. Montanier.

The author endeavors to show the great usefulness of introducing a tampon into the vagina, particularly in cases of metrorrhagia and vaginitis; he believes that the measure is attended with but little danger, and that even this could be entirely avoided by the use of some precaution; he is convinced that the usefulness of the operation, and consequently the frequency of its application, would be considerably increased if its execution could be simplified and rendered more effective. Although the introduction of charpie by means of a speculum is easy and simple enough, the removal of the same is so much the more troublesome. Gariel's caoutchouc tampons, which are inflated by air, are liable to fall out when they become lubricated by the secretions; they do not occlude the vagina completely, and do not admit the topical application of medicines. In order to remedy these defects, M. Montanier directs the tampon-ball to be covered with linen and to fasten upon this a layer of sponge, one and a half to two inches in thickness. This kind of tampon plugs up the vagina and neck of the uterus completely, without requiring to be much inflated; it never falls out and can be easily soaked with medicinal substances; moreover, it is easy to keep this tampon clean, to introduce, and to remove it. Even in dislocations of the uterus, this kind of tampon may prove very servicable.—(*Gazette des Hôpitaux*, 112, 1858.)

XIII.—*Hœmatocele Retro-Uterina Catamenialis.* By M. TROUSSEAU.

Hæmatocele retro-uterina, consists in an accumulation of blood in the peritoneal cavity in the space between the rectum and uterus, in consequence of which the uterus is displaced forward and upward. On examination, the cervix uteri is found to be pressed against the pubic bone, and behind it at the bottom of the vagina a tumor is discovered which seems to belong to the womb, and easily misleads the examiner to the diagnosis of complete retroflexion of the uterus. In the generality of cases the fundus uteri can be plainly felt and distinguished above the symphysis pubis; if, however, the effusion of blood is more considerable, and extends upward beyond the inlet of the pelvis, this cannot be done. The diagnosis can be rendered very difficult by the circumstance that a collection of blood in the cavity of the uterus exists together with the hæmatocele.

The first symptom of hæmatocele is usually pain; Nélaton explains this symptom by inflammation of the peritoneum, but the author does not agree with this view. Another symptom is paleness of the skin.

If a young woman, at the time of menstruation, suffers pain in the pelvis, accompanied by paleness of the skin, while the menstrual discharge is but very small or does not appear at all, a hæmatocele can be diagnosticated almost with certainty.

M. Trousseau considers the disease to be essentially a disorder of menstruation, a menorrhagia, which, instead of choosing the usual outlet through the uterus and vagina, finds its way into the peritoneal cavity; he believes, therefore, that the principal object of the treatment should be to regulate the disturbed menstruation. If in a still young woman the catamenial discharge is too profuse, the best remedy is cinchona, which should be given in the form of powder every three to four days in doses of about one drachm; should the remedy not be borne in this form, it may be administered in the form of enema. In an anæmic condition iron is indicated. The medication just mentioned serves to prevent further hemorrhages; but in order to master the menorrhagia at the time of its occurrence, astringents and acids should be made use of. The pain, which the author does not ascribe to inflammation of the peritoneum, but to the afflux of blood, is according to him most effectually diminished by warm poultices applied to the painful part; in recommending this measure, the author takes the view, that the increased afflux of blood is kept up by the pain, and that it is therefore necessary to endeavor first of all to remove the pain.—(*Gazette des Hôpitaux*, 75, 1858.)

XIV.—*On the Treatment of Vesico-Vaginal Fistula.* By DR. MOMM.

After Vidal and Carteaux had made the observation, that the troublesome symptoms of vesico-vaginal fistulæ are sometimes removed by the walls of the vagina growing together, the menstrual blood being then discharged through the bladder; furthermore, after Jobert, Simon, and Professor Roser, of Marburg, had obtained so much success by closing up the upper part of the vagina in such a manner as only to leave a communication between the bladder and uterus through which the menstrual blood was discharged, as in the former case, M. Simon was induced to try the successful *operation of closing up the entrance of the vagina by sutures in cases of vesico-vaginal fistulæ, which could not be removed in any other way.* Professor Roser considers the use of sutures in this operation as rather dangerous, as in sewing the bladder and the rectum together both parts have to be pierced several times with needles. In the case reported by Dr. Momm, (*Dissertation*, Marburg, 1858,) Professor Roser obtained an equally favorable result, and closed up the entrance of the vagina completely, by *making the vagina raw, and thus effecting spontaneous obliteration of this canal.* In order to make the operation successful, the vaginal mucous membrane must be of course removed rather extensively, that is, at least to the extent of one inch and a half.—(*Arch. f. Physiol. Heilkde.*, 1858.)

XV.—*On a Mechanical Impediment of Labor, not Sufficiently Noticed.* By DR. SPIEGELBERG.

More frequent than cases of real weakness of uterine contractions, are those cases in which the head, in spite of strong labor-pains, remains standing upon the bottom of the pelvis without advancing over the perineum. Both circumstances generally indicate the application of the forceps. The indication, "weakness of uterine contractions," is, however, often misused. The cause which hinders the expulsion of the head, in spite of normal labor-pains, and renders the operation with the forceps commonly very easy, is a purely mechanical one, and consists, according to *Dr. Spiegelberg, in too great flexion of the child's head upon the breast.* The face is turned toward the coccyx of the mother, the cranium rests upon the perineum, the nape of the neck is behind, the occiput below the symphysis, and in the bottom of the vagina. The uterus, already retracted above the head, acts only upon the trunk, in consequence of which the occiput is pressed more and more downward with each successive pain. On applying the forceps properly to the sides of the head and conducting it upward in the direction of the continuation of the axis of the inferior strait, the chin is removed from the breast, the neck is extended, and the head finally delivered; in order not to tear the perineum, the head must be, of course, raised but very slowly.—(*Monatschr. f. Geburtsk.*, 1858.)

